# Functional Genomics and Insights into the Pathogenesis and Treatment of Psoriasis

**DOI:** 10.3390/biom14050548

**Published:** 2024-05-03

**Authors:** Elan May Shellard, Shraddha S. Rane, Stephen Eyre, Richard B. Warren

**Affiliations:** 1 Faculty of Biology, Medicine and Health, Division of Musculoskeletal and Dermatological Sciences, School of Biological Sciences, The University of Manchester, Manchester M13 9PT, UK; 2 Centre for Genetics and Genomics Versus Arthritis, Centre for Musculoskeletal Research, NIHR Manchester Biomedical Research Centre, The University of Manchester, Manchester M13 9PT, UK; shraddha.rane@manchester.ac.uk (S.S.R.); steve.eyre@manchester.ac.uk (S.E.); 3 Dermatology Centre, Northern Care Alliance NHS Foundation Trust, Manchester M6 8HD, UK; richard.warren@manchester.ac.uk; 4 NIHR Manchester Biomedical Research Centre, Manchester University NHS Foundation Trust, Manchester Academic Health Science Centre, Manchester M23 9LT, UK

**Keywords:** psoriasis, pathogenesis, tailored medicine, functional genomics, genetics

## Abstract

Psoriasis is a lifelong, systemic, immune mediated inflammatory skin condition, affecting 1–3% of the world’s population, with an impact on quality of life similar to diseases like cancer or diabetes. Genetics are the single largest risk factor in psoriasis, with Genome-Wide Association (GWAS) studies showing that many psoriasis risk genes lie along the IL-23/Th17 axis. Potential psoriasis risk genes determined through GWAS can be annotated and characterised using functional genomics, allowing the identification of novel drug targets and the repurposing of existing drugs. This review is focused on the IL-23/Th17 axis, providing an insight into key cell types, cytokines, and intracellular signaling pathways involved. This includes examination of currently available biological treatments, time to relapse post drug withdrawal, and rates of primary/secondary drug failure, showing the need for greater understanding of the underlying genetic mechanisms of psoriasis and how they can impact treatment. This could allow for patient stratification towards the treatment most likely to reduce the burden of disease for the longest period possible.

## 1. Introduction

Psoriasis is a systemic, immune mediated, papulosquamous inflammatory skin condition, with a chronic relapsing-remitting course, which may also involve the nails and joints [1]. It affects 1–3% of the world’s population [2,3], with <0.5% being children [4]. Psoriasis occurs evenly between sexes, though early disease and increased severity are associated with being female and having an affected first-degree relative [5]. The negative impact on patients’ Health-Related Quality of Life (HRQL) causes disability comparable to major diseases such as cancer and diabetes [6]. 

Genetics are the single largest risk factor for psoriasis. Family history in psoriasis is positive for 40–50% of patients, and up to 75% in those presenting <30 years [7]. Familial clustering in psoriasis is well established, with twin studies indicating a heritability range of 70–90% [8]. HLA-C*06:02 is the main genetic risk factor for psoriasis. Inheritance of one allele increases the risk of developing psoriasis by 4–5% [9], with the IL-23/IL-17 axis, type 1 interferons, and NF-κB also having been established as key to pathogenesis [10]. This review covers the genetics of the IL-23 pathway, the movement from Genome-Wide Association Studies (GWAS) to the functional characterisation of putative risk genes, the pathogenesis of the IL-23 pathway, and its relevance to biologic and personalised treatments.

## 2. The Genetics of Psoriasis 

GWAS have identified >80 loci associated with psoriasis susceptibility in both European and east Asian populations [11], explaining up to 28% of heritability in psoriasis [12].

### 2.1. Genetic Comparison of Psoriasis Subtypes

This review covers plaque psoriasis, as it is the most common psoriasis subtype, accounting for 80–90% of patients with psoriasis. There are also, however, subtypes such as pustular psoriasis, guttate psoriasis, inverse psoriasis, and erythrodermic psoriasis. Many subsets are under-researched in comparison to the literature surrounding plaque psoriasis. Guttate, erythrodermic, and pustular forms of psoriasis have distinct morphologies, whereas other subsets are distinct from plaque psoriasis by location rather than genetics [1]. The main genes implicated in pustular psoriasis are IL36RN, CARD14, and AP1S3 [13]. Interestingly, though abnormalities in IL-36 signaling and IL-36g/a genetic polymorphisms are implicated in plaque psoriasis pathogenesis, IL36RN is not. Several loss of function mutations in IL36RN have been shown to cause generalized pustular psoriasis alone when homozygous, or heterozygous and compounded [14]. CARD14 mediates NF-κB signaling in keratinocytes; gain of function mutations here are strongly implicated in plaque psoriasis pathogenesis, whereas missense variants only seem to increase the risk of pustular psoriasis with concurrent plaque psoriasis [15,16]. AP1S3 has not been shown to have any association with plaque psoriasis, though findings have shown that loss of function mutations in AP1S3 increases the risk of pustular psoriasis independent of CARD14 and IL36RN, with Mahil et al. showing that knock out of the gene causes autophagy in keratinocytes, mediating NF-κB activation [17]. Guttate psoriasis is an outlier among subtypes, caused by a preceding streptococcal infection and often clearing within 3–4 months without treatment, though with the potential to develop into plaque psoriasis [18]. Very little is known about the pathogenesis or potential genetic basis of erythrodermic psoriasis due to its rarity, accounting for only 1–2.25% of psoriatic patients [19]. More research needs to be done to fully understand the overlap between the genetics of plaque psoriasis with the rarer psoriasis subtypes; currently, it is believed that plaque psoriasis is distinct from pustular, guttate, and erythrodermic psoriasis, though evidence also exists for some similarities to be drawn.

### 2.2. Genes Associated with the IL-23 Pathway

The identification of the IL-23R, IL12B, IL-23A, IRF4, NF-ΚBIZ, SOCS1, STAT3, and TRAF3IP2 loci (Table 1) suggests that IL-23/Th17 signaling plays a prominent role in disease pathogenesis, with IL12B coding for the p40 subunit found in both IL-23 and IL12 and TRAF3IP2 coding for ACT1, an adaptor protein essential in the signal transduction of IL17A [20]. KLF4 upregulates IL17A expression during Th17 differentiation. Significant enrichment of disease risk variants in the active chromatic domains of Th1 and Th17 cells were also found [12]. The gain of function mutation in CARD14 alone can drive IL-23/IL17 mediated psoriasiform inflammation [21]; this may be due to its role as a key mediator in the pathway through interaction with the ACT1-TRAP6 signaling complex [22], further evidenced in a study by Li et al. where epigenetic regulation of CARD14 through H3K9 demethylation controlled IL-23 expression in murine keratinocytes [23]. The loss of function mutation in IL36G increases IL36 expression, which upregulates IL6, IL-23, IL8, and NF-κB signaling [24]. TGF-β and IL-23 can increase HIF-1α expression and promote the interaction between HIF-1α and P300 in CD4+ T cells [25], leading to increased miR-210 expression in CD4+ T cells, which promotes keratinocyte proliferation, increased chemokine secretion, and increased production of TGF-β. miR-210 also promotes Th17 and Th1 cell differentiation while inhibiting Th2 differentiation by acting on STAT6 and LYN signaling [26]. 

The largest psoriasis GWAS metanalysis to date was performed by Dand et al. In 2023 [32], offering many valuable insights, with a larger sample size (36,466 cases, 458,078 controls) than previous psoriasis GWAS metanalyses [12]. With this increase in statistical power, 45 novel psoriasis susceptibility loci with genome-wide significance were identified. Of particular relevance to the IL-23 pathway, Dand et al. found a novel variant at chromosome 22q11.1, in the 5′ untranslated region/intron of IL17RA, which codes for the most common co-receptor subunit of IL-17A, IL-17C, IL-17E, and IL-17F [33]. This unit is targeted by brodalumab, a biologic found to be highly affected in the treatment of psoriasis, providing further evidence for the key nature of the IL-23 pathway [34]. 

This metanalysis also included the largest Transcriptome-Wide Association Study (TWAS) conducted in psoriasis to date. TWAS analysis correlates disease-associated Single-Nucleotide Polymorphisms (SNPs) to expression levels of genes to determine the regulatory relationship between genes and traits. TWAS focuses on modelling transcription regulation, leveraging the information garnered through expression quantitative loci (eQTL) regulation studies. In this way, it is possible to determine the level to which all disease-associated variants within a locus contribute to differential gene expression, and is a powerful tool to relate risk genotype to function. Previous TWAS studies have uncovered novel putative psoriasis risk genes [35], with Jeong et al. highlighting SSBP4 as significantly downregulated in psoriatic skin and fibroblasts [36]. SSBP4 increased transcription of interleukin 36 receptor agonist (IL36RA), IL-36RA reduces IL-36 activity, a cytokine has been found to stimulate IL-23 production and to have increased levels in psoriasis patients [37]. Dand et al. highlighted 4 genes identified or supported through the most recent TWAS: ELL, CEBPG and IRF1 and IRF5 [32]. The elongation factor gene (ELL) showed upregulation in blood with psoriasis-associated alleles, and is thought to sustain the epidermal proliferation genes known to be upregulated in psoriasis [38]. The study suggests that the CEBPG gene at a newly reported psoriasis risk locus (19q13.11), is predicted by TWAS to be downregulated in the presence of psoriasis risk variants, corroborating previous reports of C/EBPγ suppression of proinflammatory cytokines [39]. Interferon signalling has long been implicated in psoriasis pathogenesis; Dand et al. found IRF1 and IRF5 to be predicted by TWAS to be upregulated in the presence of a known psoriasis risk variant associated with IRF1 and a novel risk variant identified through the accompanying GWAS, associated with IRF5 [32].

Looking into IL-23R more specifically, Tsoi et al. identified a particularly robust susceptibility signal within this gene. The lead psoriasis associated SNP (rs9988642) is in high LD with rs11209026, a missense exonic SNP found within IL-23R. The latter SNP is protective for psoriasis, alongside other autoimmune diseases such as inflammatory bowel disease, ankylosing spondylitis, and asthma, and is present in around 7% of the population [40,41].

### 2.3. Limitations of GWAS

Index SNPs identified through GWAS are not necessarily causal and determining implicated genes in different cell types requires further analysis. Genotyped SNPs are chosen to be part of the array as they are in high Linkage Disequilibrium (LD) with many SNPs and allow identification of large genomic regions containing unmeasured SNPs who have equal probability of being causal, however they depend on cohort size and ethnicity and therefore the lead SNP can be different for different cohorts. These regions have a high probability of containing the causal SNP, however the association between a tag-SNP and a trait can be indirect, due to the tag-SNP being associated with the causal SNP [42]. 

A few risk variants are found within coding regions of genes (IL-23R, TRAF3IP2, CARD14 and IFIH1) [12], however further characterisation is required to determine the function of intronic and intergenic non-coding variants. While not coding directly for proteins, intronic variants have been found to influence gene expression through enrichment in enhancer regions [43]. Many associated SNPs are found within promotors for candidate genes and implicate that gene in disease development, such as the IL-23R, ERAP1 and IL12B loci [12]. However, the vast majority of disease-associated variants are not within coding or promoter regions, and even those that may not be implicated in disease, as seen with IL12B, where variants are intronic within RNF145, though the most likely causal gene is IL12B [44]. Intergenic variants present the greatest challenge, here the associated gene is usually postulated based on proximity and biological relevance [27]. 

### 2.4. Functional Annotation of SNPs

For intronic and intergenic SNPs, once a set of potential risk SNPS has been compiled through GWAS, bioinformatics can be used to identify SNPs in LD with the lead SNPs found through GWAS, as well as identifying alignment with histone modification or transcription factor binding sites, regulatory features that increase the likelihood of the SNPs having a causal effect. Further functional experimentation for validation of the SNPs identified as most likely to be causal can include techniques such as chromatin immunoprecipitation (ChIP), multiome single cell, CRISPR, Hi-C and eQTL in disease relevant cell types [45].

A key challenge in both bioinformatic and experimental approaches is the requirement of specific cell types, environments and stimulation to bring forth the regulatory mechanisms identified, evidenced in a variety of transcriptome studies [46,47,48]. 

RegulomeDB [49] and HaploReg [50] are databases of all known SNPs annotated with all known functional elements in a variety of cell lineages, allowing production of a score indicating the likelihood that a given SNP may be causal.

Using databases such as GTex—the most comprehensive eQTL database to date [51], eQTLs can be identified through correlation of the genomes of individuals with the expression levels of genes within specific cell types/tissues, with the lead disease-associated SNP required to correlate with the lead eQTL for strong evidence of correlation with expression. However, Fairfax et al. showed that over half of the eQTLs identified on primary monocytes were present only post-stimulation [52]. Ding J et al. built a dataset mapping eQTLs in psoriasis patient skin tissues and found significant enrichment of psoriasis GWAS SNPs—with FUT2, RPS26, and ERAP2 expression affected [53]. Although GWAS SNPs generally show significant enrichment in eQTLs [52,54,55], only 20–50% of GWAS SNPs overlap with an eQTL, and it must be noted that eQTLs prove only correlation and not causation, therefore further characterisation is required.

Laboratory based approaches can work to characterise the effect of potential causal SNPs on gene expression, alongside the mechanism of action, and relate this back to the disease phenotype. 

Capture Hi-C and HiChIP can map active chromatin interactions genome-wide with high enough resolution to identify enhancer-promoter interactions, aiding in the identification of causal genes at GWAS loci [56], as noncoding regulatory elements have been shown to interact with genes over long distances through DNA looping [57,58].

Much like eQTL, many studies have shown that chromatin interactions are cell type specific and altered during differentiation and stimulation [59,60,61], and due to the systemic nature of psoriasis, the complex interplay between skin-resident and immune cells may also play a part. ChIP, ChIP-qPCR and/or ChIP-Seq can complement these DNA-DNA interaction studies nicely, through characterisation of DNA-protein interactions at GWAS loci [62]—determining whether a potential causal allele at a risk SNP affects the level of protein binding to DNA.

The introduction of CRISPR/cas9 has had a great impact on the functional annotation of putative causal risk SNPs. This method can use fusion proteins to alter the transcriptional activity of the single SNP of interest, either activating or repressing enhancers [63,64], followed by methods such as RT-qPCR and RNA-Seq to identify differential gene expression between modified and control cells, allowing functional validation of putative causal risk SNPs. 

## 3. Pathogenesis

**Figure 1:**
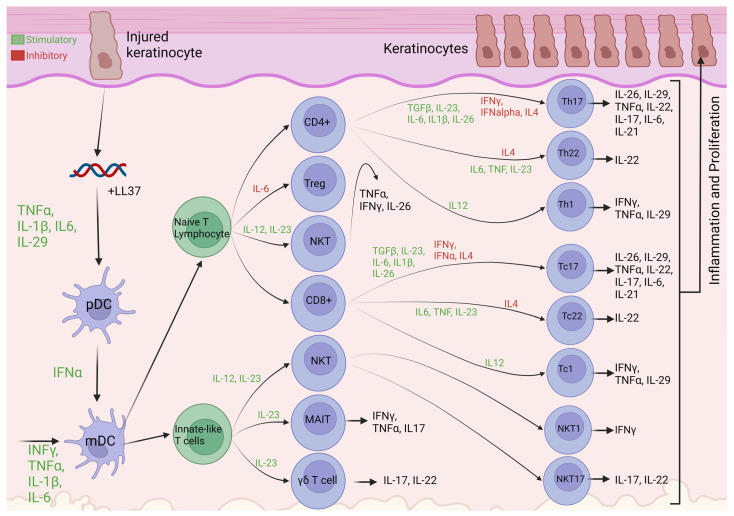
A simplified diagram of the main psoriasis pathogenesis axis. Based on majority consensus in literature; the insulted keratinocyte releases self-DNA/RNA, forming a complex with the LL37 autoantigen, which then stimulates pDCs. IFNα released by pDCs alongside cytokines released from a variety of other cells activate mDCs to go on to stimulate the differentiation of naïve T cells and innate-like T cells into mature T Cells of varying function, which go on to propagate the psoriasis phenotype. Created with BioRender.com [33,66].

The genetics show that the IL-23/Th17 pathway is key to psoriasis pathogenesis, setting it apart, alongside Crohn’s, as the only diseases to be so, with other immune mediated diseases being mainly Treg/Th1 driven. Figure 1 shows a simplified version of the psoriasis axis: When a keratinocyte is injured due to illness, infection or environmental reasons, it released self-DNA/RNA, which forms a complex with the LL37 autoantigen, these complexes activate both myeloid dendritic cells (mDCs) to produce TNF-α, IL-23, IL-12 and plasmacytoid dendritic cells (pDCs) to produce IFN-α via stimulation of TLR9, TLR7 and TLR8, this leads to the activation and migration to the lymph nodes of local mDCs (also known as conventional dendritic cells), which can activate T cells through antigen presentation [65]. mDCs are also activated by INF-γ, TNF-α, IL-1-β, and IL-6 secreted by innate immune cells such as keratinocytes, macrophages and natural kill (NK) T cells (Table 2), and go on to produce TNF-α, IL-12, IFNα and -β, IL-6 and IL-23, these cytokines then cause the differentiation and proliferation of naïve T lymphocytes to varying T cells including T helper (Th) 17 and Th22 lymphocytes, which move into the blood and skin. Th17 lymphocytes release IL-17 alongside γδ T lymphocytes, NK cells, mastocytes, and innate lymphoid cells (ILCs), as well as IL-22, IFNγ, IL-2 and IL-29, whereas Th22 lymphocytes release IL-22 alone. IL22, IL17a and IL17f cause development of the psoriasis phenotype through the proliferation and impaired differentiation of keratinocytes. This process also includes many mechanisms of positive feedback, causing propagation of the disease and increasing inflammation [33,66].

A key cell type seen in Figure 1, dendritic cells (DCs) provide the link between innate and adaptive immunity, and in psoriasis this manifests as the link between disease initiation and propagation. Studies have shown increased pDC infiltration in psoriasis vs healthy skin [86], pDCs usually have safeguards against recognition of self-nucleic acids, however the large amounts of antimicrobial peptides such as LL-37 produced in psoriasis enables their recognition, leading to the production of vast amounts of IFNα [73]. pDCs are the main producers of IFNα in the skin. Nestle et al. (2005) and Okada et al. (2014) previously determined the importance of IFNα in the development of the psoriasis phenotype [87,88]. This stance is supported by genetic analysis revealing DDX58 and RNF114, both type 1 IFN genes, to confer psoriasis risk. IFNα also stimulates the differentiation of monocytes into inflammatory dendritic cells (iDCs) and CD4+ T cells into Th1 and Th17 cells [89]. iDC levels are reported to be increased in psoriasis and have been shown to present antigens to CD4+ helper and CD8+ cytotoxic cells and produce cytokines such as IL-12, IL-23, TNF-α, IL-1β, IL-6 and TGF-β [73]. 

There are two types of conventional dendritic cells, also known as myeloid dendritic cells (mDCs). Type 1 mDCs are known as resident dendritic cells, and are antigen presenting cells (APCs) that present to T lymphocytes, they are BDCA-1-positive (CD1c+), and numbers are normal in psoriasis [90]. Type 2 mDCs are BDCA-1-negative (CD1c−), with numbers greatly increased in psoriasis lesions, and normalising after treatment with biologics. Also known as inflammatory DCs or TiP-DCs, type 2 mDCs produce TNF-α, inducible nitric oxide synthase (iNOS), IL-6, IL-12, IL-20, and IL-23 [73], and again link the innate and adaptive immune systems through stimulation of naïve T cell to differentiate and presentation of foreign antigens to CD8+ T cells through cross presentation [91]. mDCs can also be directly stimulated by TNFα and LL37-self nuclease complexes [73].

### Main Intracellular Pathways

The IL-23 protein itself is key to the IL-23/Th17 axis, stimulating differentiation of Th22 and Th17 cells, release of inflammatory cytokines and feeding the positive feedback loop propagating inflammation within psoriatic plaques.

IL-23 is a heterodimeric complex of p19 and p40 subunits, p19 is shared with IL-39, whereas the p40 subunit is found in IL-12. The receptor for IL-23 consists of IL-12Rβ1, shared with IL-12, and an IL-23Rα chain. This structural similarity with IL-12 along with IL-12s possible protective role in psoriasis greatly influenced the development of biologics aimed to target the p19 subunit specifically (Table 3) [69,92]. A study by Lee et al. also found that the expression of both p19 and p40 subunits was upregulated in psoriasis, as opposed to the IL-12 specific p35 [93]. 

In disease status, the JAK/STAT3 pathway is activated by INF-γ, IL-12, IL-22, and IL-23 (Table 2) [66,68]. The binding of IL-23 to IL-23R attracts a heterodimer of JAK2 and TYK2, which binds to its intracellular domain. The heterodimer then auto-phosphorylates, which activates the receptor and attracts STAT proteins, which bind and are phosphorylated before moving to the nucleus to regulate gene transcription [104]. TYK2 specifically is mainly activated by IL12 and IL-23—the lead receptor dimerises IL-12Rβ1/IL-23R, IL-12Rβ1 associates with Tyk2 and its heterotypic subunits, while IL-23R binds to Jak2. TYK2 deficiency leads to reduced ability to recruit Th17 and Th22 cells [105]. STAT3 is hyperactivated in immune cells and keratinocytes, inhibits cell differentiation, and promotes proliferation and production of antimicrobial proteins (AMPs) in response to IL-23, IL-6, IL-17, IL-21, IL-19 and IL-22 [33]. STAT3 is activated by phosphorylation of a conserved tyrosine residue by JAK kinases [68]. Phosphorylated STAT3 enhances RORγt expression, an intracellular regulator for the proliferation and function of Th17 cells [106], and both bind to promoters of genes such as IL17A, IL17F, IL22, IL26, and IL-23R [94]. STAT3 mediates the effects of IL-23, so is essential for the amplification and maintenance of Th17 differentiation, it upregulates IL17A and F expression, alongside other genes required for the Th17 pathway, such as RORγT, RORα, BATF, IRF4, AHR, IL-6Rα, and C-MAF, as well as being essential for the function of γδ T cells (Calautti et al., 2018). STAT3 also inhibits the convergence of Tregs downstream of IL6 and IL-23 signaling, leading to a loss in suppressive power, as well as mediating IL6 stimulated IL21 secretion by naïve T cells, leading to the induction of IL-23R and IL27 expression [68]. 

RORγt is a nuclear receptor required for Th17 cell differentiation from both murine and human CD4+ T cells. Stimulated by IL-23 and IL6, it acts on Th17 gene promoters IL17A, IL17F, IL22, IL26, IL-23R, Csf-2, CCR6, and CCL20. Success of IL-23 targeted biologics, and studies showing that lack of RORγt leads to failure of Th17 cells to differentiate demonstrates its potential as an effective drug target [106,107].

NF-κB is formed of a group of proteins, including RelA (p65), RelB and c-Rel, together with subunits of NF-κB1 (p105) and NF-κB2 (p100), processed into p50 and p52 (Perkins et al., 1992), it forms dimers, though these are retained in the cytoplasm by IκB proteins. NF-κB signalling is induced by many inflammatory cytokines (Table 2) leading to the phosphorylation of IκBα by IKKβ, degradation of IκB through proteins such as TRAFs and ACT1, and phosphorylation of IKKs for translocation to the nucleus to regulate transcription [108]. Many psoriasis risk genes are involved in this pathway; TNFAIP3, NF-ΚBIZ and TNIP1 are involved in pathway regulation, with NF-ΚBIA inhibiting the pathway, RELA coding for an NF-κB subunit and TRAF3IP2 coding for ACT1 (Table 1). Inhibition of NF-κB signaling has been shown to decrease levels of IL-23 mRNA [109]. 

Looking specifically at intracellular signaling, genes associated with the signaling pre and post IL-23 production are implicated in psoriasis GWAS. Interestingly, Lysell et al. found that 5 SNPs within the IL-23R, IL-23A and IL12B genes were only associated with severe psoriasis, alongside a significant difference in NF-ΚB1 when stratifying the cohort based on disease severity. TYK2 also showed higher expression in the severe cohort, with the association disappearing in the milder group. Out of the determined risk genes, only STAT3, TNFAIP3 and TRAF3IP2 associations remained significant in all groups, with no significant difference between disease severities. Most interestingly, interaction between genes associated with the NF-κB pathway and IL-23 signaling was increased in the severe phenotype group, with interaction between risk alleles in IL-23R, NF-ΚB1, TNIP1, IL12B, and IL-23A only seen in the severe cohort [110]. This study is interesting and provides some support for the link between NF-κB signaling and IL-23 production and downstream signaling shown in Table 2; however, it is the only study on this topic and so requires further validation. 

## 4. Biologic Treatments

Patient response in psoriasis is commonly measured using the Psoriasis Area and Severity Index (PASI). PASI is calculated through clinician assessment of the percentage body area affected with psoriasis and the severity of each area impacted. The score can range from 0–72, generally a score of 5–10 is considered moderate disease and >10 as severe. A 75% or 90% reduction in PASI is the benchmark in most clinical trials, noted as PASI75 and PASI90, respectively [111]. 

Comparing currently available psoriasis biologics (Table 3); in TNFα inhibitors, etanercept is barely superior against systemic treatment options [112], though infliximab and adalimumab performed better [113,114]. Although superior to etanercept, ustekinumab was inferior to all IL-17 therapeutics, due to lower specificity and the possible protective effect of IL-12 [115]. Risankizumab and guselkumab have proved superior to ustekinumab and TNF inhibitors, with tildrakizumab being the least successful IL-23p19 antagonist, possibly due to lower affinity [96,116,117]. There is similarity in efficacy between IL-17 and IL-23p19 antagonists, with ixekizumab having a faster response, possibly due to IL-23p19 inhibitors acting further upstream, but guselkumab having the better long-term result [97,118]. The recently approved bimekizumab works at a faster rate and, based on network meta-analysis, seems to be one of the highest performing biologics to date [102], possibly due to its inhibition of both IL-17A and F, whereas IL-23 inhibitors allow for the production of IL-17 through other mechanisms. However, it has yet to be compared to risankizumab or guselkumab. 

### 4.1. Biologic Efficacy in Psoriasis 

As observed commonly with biologics, patients’ initial response tapers off over time (secondary failure) though some do not respond at all (primary failure). The time between first response and withdrawal of the drug due to loss of efficacy differs between biologics, though the risk of treatment failure is positively correlated with the number of biologics the patient has previously tried [119]. A 2022 study by Elberdín et al. [120] found that over 10 years, the median number of biologics patients had been on was 2 (range 1–8), with lack of efficacy being the main reason for switching. It found that ustekinumab had the best drug survival, with efalizumab being withdrawn from the market in 2009 (Table 3). As IL-23p19 inhibitors show an increased remission period post drug withdrawal compared to ustekinumab, it will be interesting to see whether it would have increased survival in 10 years. The mechanisms leading to treatment failure remain unclear. 

One possible reason could be the development of antidrug antibodies (ADAs). Specific to biologic treatments, an immune response can be generated to target the monoclonal antibodies, leading to reduced circulating drug levels, drug efficacy, drug survival and/or adverse effects such as infusion reactions [121]. A possible solution is the administration of immunosuppressants alongside biologic treatment, such as methotrexate/azathioprine co-prescription with TNF inhibitor treatments, though this does come with the risk of immunosuppression in patients [121]. Interestingly, the development of ADAs can be influenced by genetic factors, with the HLA-DRβ-11, HLA-DQ-03, and HLA-DQ-05 alleles conferring a higher risk of ADA development post anti-TNF treatment [122]. The most consistent genetic association with ADA development is HLA-DQA1*05 alleles, however the relatively small sample sizes and number of associations, and lack of consistent result replication found in these studies make drawing reliable conclusions difficult [123,124,125].

Another possible mechanism is genetic polymorphisms. With response to biologic drugs typically being heterogenous, one hypothesis is that this response reflects genetic variance between patients or genetically distinct disease subsets with distinct pathogeneses. The effect of genetics on anti-TNF response is well characterized, with TNF-α, TNFRSF1A, TNFRSF1B, TNFAIP3, FCGR2A, FCGR3A, IL-17F, IL-17R, and IL-23R suggested to modulate response [126], however, few studies explore the interaction of IL-17 and IL-23 inhibitors with genetics. Ustekinumab shows a higher efficacy and faster response time in HLA-Cw*06 positive patients than in negative patients [126], and Van den Reek et al. found that the IL12B rs3213094-T allele increased efficacy and TNFAIP3 rs610604-G allele predicting a worse outcome [127], however other studies were unable to replicate this. The SUPREME study found that HLA-Cw*06 status did not influence response to secukinumab [128], however the two Italian studies predicted a higher PASI90 in HLA-Cw*06 positive patients [129,130]. An investigation into the effects of IL-17A polymorphisms on secukinumab and ixekinumab response identified five non-coding SNPs, however none influenced PASI75/90 achievement at 12 or 24 weeks [131]. In conclusion, a link between genetic and treatment response has been found, however, especially in regard to the newer and more effective IL-17 and IL-23 inhibitors, more studies are needed to reliably determine the effects of the polymorphisms identified as modulating treatment response. Discovery of genetic biomarkers for drug response could allow stratification of patients into subgroups to increase response rates, allowing patients an earlier increase in quality of life.

### 4.2. Biologic Withdrawal in Psoriasis 

Patients withdraw from therapeutics for a variety of reasons; withdrawal is associated with risk of relapse, though time to relapse varies between person and drug. The median time to relapse was 16–20 weeks for tildrakizumab, an IL-23p19 inhibitor (defined as below PASI 90), or 20–25 weeks for PASI < 75 [132], whereas guselkumab had a median relapse time of 15 weeks post withdrawal (PASI < 90) [97] and risankizumab a median of 30 weeks (PASI 90) [133]. Ustekinumab’s median time was 15 weeks to PASI < 75 post withdrawal, 22–24 weeks for PASI < 50 [134,135]. While IL17 inhibitors seem to have a shorter time to relapse and occasional rebound of disease, studies conflict over median time, from 46 days (brodalumab) [136] to 20 weeks (ixekizumab, PASI < 50) [137], this difference is likely due to differing relapse criteria. The median time to relapse when withdrawing TNFα inhibitors has been found as 12.1 weeks (etanercept) [138] to 19.5 weeks (infliximab) [139], the shortest post-withdrawal period [140]. The increased time period for IL-23 inhibitors may because IL-23 is an upstream cytokine of many psoriasis pathways, impacting cytokines such as IL17, and potentially the proliferation and survival of epidermal T cells [132]. 

A recent study published by Zhang et al. focused on secukinumab, which targets genes thought to confer psoriasis risk both upstream (IL-23R, TYK2, JAK2, STAT3) and downstream (TRAF3IP2/ACT1, TNFAIP3/A20) of IL-17 production. They found that although genetic variation in the IL-17 pathway impacts psoriasis susceptibility, this same variation does not significantly impact treatment response to secukinumab [141]. However, due to possible conflict of interest, further studies in this subject would be useful.

Together with the highlighted importance of genetics in understanding and determining psoriasis pathogenesis, this review emphasises the need for the use of genetics to stratify patients towards treatment options that are most likely reduce disease burden for the longest period possible, as currently there is no tool or technique in the choice of first biologic, or those that follow, past clinician experience and preference.

## 5. Summary

There have been enormous advances in the genetic understanding of the risk to developing psoriasis. Similar to Crohn’s disease, there is large and growing evidence as to the importance of the IL-23/IL17 pathway in disease. Risk variants are now more reliably linked to causal genes through functional genomic technologies and bioinformatics, providing a better understanding of the key genes, pathways, and cells types in disease.

Biologic therapies have transformed psoriasis treatment, targeting specific immune pathways. However, individual responses vary, prompting a focus on genetic factors influencing treatment outcomes. Recent studies highlight genetic polymorphisms’ role in treatment response, particularly in inflammatory pathway genes like TNF-α, IL-17, and IL-23R. Further studies into the effects of risk alleles on treatment response are required, as current knowledge suggests potential for personalized treatment selection.

Here, we have brought together a variety of disciplines necessary to translate identification of possible risk SNPS through computational means, to the characterization of putative causal genes in the lab, and ending with the clinical benefit to the psoriasis patient. Integrating genetic data into treatment decisions offers promise for personalized psoriasis management. Continued research will refine our understanding and optimize treatment approaches for better patient outcomes.

## Figures and Tables

**Table 1:** Non-MHC GWAS loci associated with increased risk of psoriasis. The risk of single-nucleotide polymorphisms (SNPs) identified through various psoriasis GWAS and the potentially associated genes, with genes identified as relevant to the IL-23 pathway highlighted. Adaption of table from Ray-Jones et al. 2018 [27].

Locus	Notable Gene(s) in Literature	Study Population	Index SNP	Index SNP Annotation	*p*-Value	Reference
1p36.3	*MTHFR*	CHN	rs2274976	Missense: *MTHFR*	2.33 × 10^−10^	
1p36.23	*SLC45A1, TNFRSF9*	EUR	rs11121129	Intergenic	1.7 × 10^−8^	
1p36	*IL-28RA*	EUR	rs7552167	4.2 kb 5′ of *IL-28RA*	8.5 × 10^−12^	
		CHN	rs4649203	5.5 kb 5′ of *IL-28RA*	9.74 × 10^−11^	
1p36.11	*RUNX3*	EUR	rs7536201	1.5 kb 5′ of *RUNX3*	2.3 × 10^−12^	
1p36.11	*ZNF683*	CHN	rs10794532	Missense: *ZNF683*	4.18 × 10^−8^	
1p31.3	*IL-23R*	EUR	rs9988642	441 bp 3′ of *IL-23R*	1.1 × 10^−26^	
		CHN	chr1: 67,421,184 (build hg18)	Nonsynonymous: *IL-23R*	1.94 × 10^−11^	
1p31.3	*C1orf141*	CHN	rs72933970	Missense: *C1orf141*	1.23 × 10^−8^	
1p31.1	*FUBP1*	EUR	rs34517439	Intronic: *DNAJB4*	4.43 × 10^−9^	
1q21.3	*LCE3B, LCE3D*	EUR	rs6677595	3.6 kb 3′ of *LCE3B*	2.1 × 10^−33^	
		CHN	rs10888501	175 bp 3′ of *LCE3E*	6.48 × 10^−13^	
1q22	*AIM2*	CHN	rs2276405	Stop-gained: *AIM2*	3.22 × 10^−9^	
1q24.3	*FASLG*	EUR	rs12118303	Intergenic	3.02 × 10^−10^	
1:24964519	*RUNX3*	JAP	rs6672420	Missense: *RUNX3*	7 × 10^−10^	[28]
1p36.22	*MTHFR*	S.ASIAN/EUR	rs2103876	Intronic: *MFN2*	1.18 × 10^−9^	[29]
1q24.2	*XCL1*	S.ASIAN/EUR	rs12046909	3′ of gene: *XCL2*	1.68 × 10^−9^	[29]
1q31.1	*LRRC7*	EUR	rs10789285	Intergenic	1.43 × 10^−8^	
1q31.3	*DENND1B*	EUR	rs2477077	Intronic: *DENND1B*	3.05 × 10^−8^ (meta)	
1q32.1	*IKBKE*	EUR	rs41298997	Intronic: *IKBKE*	2.37 × 10^−8^	
2p16.1	*FLJ16341, REL*	EUR	rs62149416	Intronic: *FLJ16341*	1.8 × 10^−17^	
2p15	*B3GNT2*	EUR	rs10865331	Intergenic	4.7 × 10^−10^	
2q12.1	*IL1RL1*	CHN	rs1420101	Intronic: *IL1RL1*	1.71 × 10^−10^	
2q24.2	*KCNH7, IFIH1*	EUR	rs17716942	Intronic: *KCNH7*	3.3 × 10^−18^	
		CHN	rs13431841	Intronic: *IFIH1*	2.96 × 10^−9^	
2:60847551	*REL-DT*	JAP	rs1177203	Intronic: *REL-DT*	4 × 10^−9^	[28]
3p24.3	*PLCL2*	EUR	rs4685408	Intronic: *PLCL2*	8.58 × 10^−9^	
3q11.2	*TP63*	EUR	rs28512356	400 bp 3′ of *TP63*	4.31 × 10^−8^	
3q12.3	*NF-ΚBIZ*	EUR	rs7637230	Intronic: RP11-221J22.1	2.07 × 10^−9^	
3:101914516	*RDUR, NFKBIZ*	JAP	rs2312786	Intronic	1 × 10^−9^	[28]
3q13	*CASR*	CHN	rs1042636	Missense: *CASR*	1.88 × 10^−10^	
3q26.2-q27	*GPR160*	CHN	rs6444895	Intronic: *GPR160*	1.44 × 10^−12^	
4q24	*NF-ΚB1*	CHN	rs1020760	Intronic: *NF-ΚB1*	2.19 × 10^−8^	
4:105719474	*INTS12,GSTCD*	CHN	rs149442660	Intronic: *INTS12*, Missense: *GSTCD*	6 × 10^−12^	[30]
4:121833304	*BBS7*	CHN	rs143700362	Missense: *BBS7*	3 × 10^−19^	[30]
5p13.1	*PTGER4, CARD6*	EUR	rs114934997	Intergenic	1.27 × 10^−8^	
5q14	*ZFYVE16*	CHN	rs249038	Missense: *ZFYVE16*	2.14 × 10^−8^	
5q15	*ERAP1, LNPEP*	EUR	rs27432	Intronic: *ERAP1*	1.9 × 10^−20^	
		CHN	rs27043	Intronic: *ERAP1*	6.50 × 10^−12^	
5q31	*IL13, IL4*	EUR	rs1295685	3′-UTR: *IL13*	3.4 × 10^−10^	
5q33.1	*TNIP1*	EUR	rs2233278	5′-UTR: *TNIP1*	2.2 × 10^−42^	
		CHN	rs10036748	Intronic: *TNIP1*	4.26 × 10^−9^	
5:151087628		JAP	rs2233278	5′-UTR: *TNIP1*	3.7 × 10^−10^	[31]
5q33.3	*IL12B*	EUR	rs12188300	Intergenic	3.2 × 10^−53^	
		CHN	rs10076782	Intronic: *RNF145*	4.11 × 10^−11^	
5:159402519	*IL12B, LINC01845*	JAP	rs12188300	Intronic	3 × 10^−23^	[28]
5:151090412	*TNIP1*	JAP	rs74817271	Intronic: *TNIP1*	6 × 10^−15^	[28]
5q33.3	*PTTG1*	CHN	rs2431697	Intergenic	1.11 × 10^−8^	
6p25.3	*EXOC2, IRF4*	EUR	rs9504361	Intronic: *EXOC2*	2.1 × 10^−11^	
6p22.3	*CDKAL1*	EUR	rs4712528	Intronic: *CDKAL1*	8.4 × 10^−11^	
6:31014767	*MUC22*	JAP	rs9394026	Intronic: *MUC22*	6.6 × 10^−15^	[28]
6:31271729	*HLA-C*	JAP	rs1050414	Synonymous: *HLA-C*	6 × 10^−14^	[28]
6:108049381	*OSTM1*	CHN	rs149798287	Missense: *OSTM1*	1 × 10^−8^	[30]
6:111608659	*TRAF3IP2, FYN*	JAP	rs9481169	5′ of *TRAF3IP2*	7 × 10^−12^	[28]
6:137918297	*SIMALR, TNFAIP3*	JAP	rs6933987	Intergenic	2 × 10^−8^	[28]
6:159094277	*TAGAP-AS1, FNDX1-AS1*	JAP	rs2249937	Intronic	1 × 10^−11^	[28]
6:31333042	*HLA-B, LINC02571*	JAP	rs12212594	Intergenic	5 × 10^−209^	[28]
6q23.3	*TRAF3IP2*	EUR	rs33980500	Missense: *TRAF3IP2*	4.2 × 10^−45^	
	*TNFAIP3*	EUR	rs582757	Intronic: *TNFAIP3*	2.2 × 10^−25^	
6q25.3	*TAGAP*	EUR	rs2451258	Intergenic	3.4 × 10^−8^	
7p14.3	*CCDC129*	CHN	rs4141001	Missense: *CCDC129*	1.84 × 10^−11^	
7:141614171	*AGK*	CHN	rs144706178	Missense: *AGK*	2 × 10^−15^	[30]
7p14.1	*ELMO1*	EUR	rs2700987	Intronic: *ELMO1*	4.3 × 10^−9^	
8p23.2	*CSMD1*	CHN	rs10088247	Intronic: *CSMD1*	4.54 × 10^−9^	
9p21.1	*DDX58*	EUR	rs11795343	Intronic: *DDX58*	8.4 × 10^−11^	
9q31.2	*KLF4*	EUR	rs10979182	Intergenic	2.3 × 10^−8^	
10q21.2	*ZNF365*	EUR	rs2944542	Intronic: *ZNF365*	1.76 × 10^−8^	
10q22.2	*CAMK2G, FUT11*	EUR	rs2675662	Intronic: *CAMK2G*	7.35 × 10^−9^	
10q22.3	*ZMIZ1*	EUR	rs1250544	Intronic: *ZMIZ1*	3.53 × 10^−8^	
10:88732445	*LIPK*	CHN	rs200583975	Missense: *LIPK*	1 × 10^−7^	[30]
10q23.31	*PTEN, KLLN, SNORD74*	EUR	rs76959677	Intergenic	2.75 × 10^−8^	
10q24.31	*CHUK*	EUR	rs61871342	Intronic: *BLOC1S2*	1.56 × 10^−9^	
11p15.4	*ZNF143*	CHN	rs10743108	Missense: *ZNF143*	1.70 × 10^−8^	
11q13	*RPS6KA4, PRDX5*	EUR	rs694739	256 bp 5′ of AP003774.1	3.71 × 10^−9^	
11q13.1	*CFL1, FIBP, FOSL1*	EUR	rs118086960	Intronic: *CFL1*	6.89 × 10^−9^	
11q13.1	*AP5B1*	CHN	rs610037	Synonymous: *AP5B1*	4.29 × 10^−11^	
11q22.3	*ZC3H12C*	EUR	rs4561177	1.7 kb 5′ of *ZC3H12C*	7.7 × 10^−13^	
11q24.3	*ETS1*	EUR	rs3802826	Intronic: *ETS1*	9.5 × 10^−10^	
12p13.3	*CD27, LAG3*	CHN	rs758739	Intronic: *NCAPD2*	4.08 × 10^−8^	
12p13.2	*KLRK1, KLRC4*	EUR	rs11053802	Intronic: *KLRC1*	4.17 × 10^−9^	
12q13.3	*IL-23A, STAT2*	EUR	rs2066819	Intronic: *STAT2*	5.4 × 10^−17^	
12q24.12	*BRAP, MAPKAPK5*	EUR	rs11065979	Intergenic	1.67 × 10^−8^	
12q24.31	*IL31*	EUR	rs11059675	Intronic: *LRRC43*	1.50 × 10^−8^	
13q12.11	*GJB2*	CHN	rs72474224	Missense: *GJB2*	7.46 × 10^−11^	
13q14.11	*COG6*	EUR	rs34394770	Intronic: *COG6*	2.65 × 10^−8^	
13q14.11	*LOC144817*	EUR	rs9533962	Within LOC144817	1.93 × 10^−8^	
13q32.3	*UBAC2, RN7SKP9*	EUR	rs9513593	Intronic: *UBAC2*	3.60 × 10^−8^	
14q13.2	*NF-ΚBIA*	EUR	rs8016947	Intronic: RP11-56B11.3	2.5 × 10^−17^	
13q14.11	*LOC144817*	CHN	rs12884468	Intergenic	1.05 × 10^−8^	
14q23.2	*SYNE2*	CHN	rs2781377	Stop-gained: *SYNE2*	4.21 × 10^−11^	
14q32.2	*RP11-61O1.1*	EUR	rs142903734	Intronic: *RP11-61O1.1*	7.15 × 10^−9^	
15q13.3	*KLF13*	EUR	rs28624578	Intronic: *KLF13*	9.22 × 10^−10^	
16p13.13	*PRM3, SOCS1*	EUR	rs367569	1.6 kb 3′ of *PRM3*	4.9 × 10^−8^	
16p11.2	*FBXL19, PRSS53*	EUR	rs12445568	Intronic: *STX1B*	1.2 × 10^−16^	
17q11.2	*NOS2*	EUR	rs28998802	Intronic: *NOS2*	3.3 × 10^−16^	
17q12	*IKZF3*	CHN	rs10852936	Intronic: *ZPBP2*	1.96 × 10^−8^	
17q21.2	*PTRF, STAT3, STAT5A/B*	EUR	rs963986	Intronic: *PTRF*	5.3 × 10^−9^	
17q25.1	*TRIM47, TRIM65*	EUR	rs55823223	Intronic: *TRIM65*	1.06 × 10^−8^	
17q25.3	*CARD14*	EUR	rs11652075	Missense: *CARD14*	3.4 × 10^−8^	
17q21.2	*PTRF, STAT3, STAT5A/B*	CHN	rs11652075	Missense: *CARD14*	3.46 × 10^−9^	
17q25.3	*TMC6*	CHN	rs12449858	Missense: *TMC6*	2.28 × 10^−8^	
18p11.21	*PTPN2*	EUR	rs559406	Intronic: *PTPN2*	1.19 × 10^−10^	
18q21.2	*POL1, STARD6, MBD2*	EUR	rs545979	Intronic: *POL1*	3.5 × 10^−10^	
18q22.1	*SERPINB8*	CHN	rs514315	3′ of *SERPINB8*	5.92 × 10^−9^	
19p13.2	*TYK2*	EUR	rs34536443	Missense: *TYK2*	9.1 × 10^−31^	
19p13.2	*ILF3, CARM1*	EUR	rs892085	Intronic: *QTRT1*	3 × 10^−17^	
19:10366391	*TYK2*	JAP	rs34725611	Intronic: *TYK2*	4 × 10^−13^	[28]
19:4862608	*SPHK*	CHN	rs11544355	Missense: *SPHK*	7 × 10^−11^	[30]
19q13.33	*FUT2*	EUR	rs492602	Synonymous: *FUT2*	6.57 × 10^−13^	
19q13.41	*ZNF816A*	CHN	rs12459008	Missense: *ZNF816*	2.25 × 10^−9^	
20q13.13	*RNF114*	EUR	rs1056198	Intronic: *RNF114*	1.5 × 10^−14^	
21q22	*RUNX1*	EUR	rs8128234	Intronic: *RUNX1*	3.74 × 10^−8^	
21q22.11	*IFNGR2*	CHN	rs9808753	Missense: *IFNGR2*	2.75 × 10^−8^	
21q22.11	*SON*	CHN	rs3174808	Missense: *SON*	1.15 × 10^−8^	
22q11.21	*UBE2L3, YDJC*	EUR	rs4821124	1 kb 3′ of *UBE2L3*	3.8 × 10^−8^	

**Table 2:** A summary of cell types involved in the IL-23 pathway in psoriasis. This table summarises the cell types immediately involved in an IL-23 driven psoriasis pathway, illustrating the relevant stimulants responded to, intracellular pathways activated, and proteins produced.

Cell Type	Stimulant	Intracellular Signalling	Production	References
Keratinocyte	TNFα	NFκB	IL-23	[33,66,67]
	IL-17	ACT1/TRAF6 NFκB/MAPK	IL-23	
	IL-36	MyD88/IRAK/MAPK/NFκB	IL-23	
	IL-23	JAK/STAT3	CCL20	
			TGFβ	
Th17	IL-36	MyD88/IRAK/MAPK/NFκB	IL-23	[67,68]
	IL-23	JAK/STAT3	IL-17	
			IL-22	
			IFNγ	
			IL-2	
			IL-29	
ILC3	IL-23	JAK/STAT3	IL-23	[67,69]
			IL-17	
Monocytes	Mycobacterium	NFκB	IL-23	[67]
	IL-23	JAK/STAT3	IL-22	
Macrophage	IFNγ	JAK/STAT1	IL-23	[70,71]
	Microbial infection	Dependent on microbe	IL-23	
	IL-23	JAK/STAT3	Increased IL-23R expression	
			TNFα	
	IL-36γ	MyD88/IRAK/MAPK/NFκB	IL-23	
IL-23 Macrophage	IL-23	JAK/STAT3	IL-17A/F	[72]
			IL-22	
			IFNγ	
Myeloid dendritic cell	IFNα	JAK/STAT1/2	IL-23	[73]
	IFNγ	JAK/STAT1	IL-23	
	TNFα	NFκB	IL-23	
Langerhans cell	IL-36γ	MyD88/IRAK/MAPK/NFκB	IL-23	[73]
Skin resident memory T cells	IL-23	JAK/STAT3	Proliferation	[74,75]
			IL-17	
Naïve T cell	IL-23	JAK/STAT3	Inhibition of Treg convergence	[76]
Th1	IL-23	JAK/STAT3	IFNγ	[67]
			IL-26	
			IL-17	
			IL-22	
			IL-29	
Th22	IL-23	JAK/STAT3	IL-22	[77]
Neutrophil	IL-23	JAK/STAT3	IL-17	[69,78]
			LL36	
			Extracellular trap formation	
Treg	IL-23	JAK/STAT3	IFNγ	[79]
			TNFα	
			IL-17A	
γδ T cell	IL-23	JAK/STAT3	IL-17	[80,81,82]
			IL-22	
αβ T cell	IL-23	JAK/STAT3	IL-17	[83]
NK22	IL-23	JAK/STAT3	IL-22	[66,69]
NK17	IL-23	JAK/STAT3	Differentiation	
			IL-17	
			IFNγ	
NKT1	IL-23	JAK/STAT3	IFNγ	[84]
MAIT17 cells	IL-23	JAK/STAT3	IL-17	[85]

**Table 3:** Summary of biologic drugs used in psoriasis treatment. This table summarises the biologic drugs used in psoriasis treatment, alongside their targets and mechanisms of action.

Drug	Target	Mechanism	References
Ustekinumab	P40 subunit shared by IL12 and IL-23	Disrupts Th1 and Th17 differentiation and IL12 and IL-23 signaling	[94,95]
Guselkumab Tildrakizumab Risankizumab	P19 subunit of IL-23	Disrupt Th17 and IL-23 signaling	[69,96,97,98,99]
Secukinumab Ixekinumab	IL17A	Prevents both IL17A homodimers and IL17a-IL17F heterodimers binding to their receptors.	[34,69,94,100,101]
Brodalumab	IL17RA	Due to the commonality of the IL17RA chain in receptor complexes, interrupts signaling of IL-17A, IL-17C and IL-17F homodimers and the IL-17A/F heterodimer	
Bimekizumab	IL17A/F	Prevents IL17A and F homodimers and the IL17A-IL17F homodimer binding to their receptors.	[102]
Etanercept Adalimumab Infliximab Certolizumab	TNF-α	Indirect impact on IL17, by regulation of IL-23 production from myeloid or CD11c+ dendritic cells.	[69,94,103]
